# Rapid and ultrasensitive detection of *Candida tropicalis* using multiple cross-displacement amplification combined with CRISPR/Cas12a

**DOI:** 10.3389/fcimb.2026.1796886

**Published:** 2026-07-30

**Authors:** Rui Ye, Mao Liu, Xujian Zhang, Qi Liang, Yumei Cao, Chuiyu Zhu, Honglan Yu, Shu Zhang, Yu Wang

**Affiliations:** 1 Department of Basic Clinical Laboratory Medicine, School of Clinical Laboratory Science, Guizhou Medical University, Guiyang, Guizhou, China; 2 Department of Clinical Laboratory, The First People’s Hospital of Guiyang, Guiyang, Guizhou, China; 3 Center for Clinical Laboratories, The Affiliated Hospital of Guizhou Medical University, Guiyang, Guizhou, China

**Keywords:** *Candida tropicalis*, CRISPR/Cas12a, invasive fungal disease, multiple cross displacement amplification, ultrasensitive detection

## Abstract

**Background:**

*Candida tropicalis* (*C. tropicalis*), classified as a World Health Organization “critical priority” pathogen, causes life-threatening invasive infections with high mortality due to diagnostic delays. Therefore, the development of rapid fungal detection platforms is an urgent scientific and clinical priority.

**Methods:**

We designed multiple cross-displacement amplification (MCDA) primers to target the internal transcribed spacer 2 (ITS II) gene of *C. tropicalis* for specific amplification. Subsequently, the clustered regularly interspaced short palindromic repeats (CRISPR)/Cas12a-crRNA complex bound to the amplified products, activating its trans-cleavage activity to generate a detectable fluorescent signal. Finally, clinical samples were used to validate the detection results, which were further compared with those obtained using fungal culture and multiplex polymerase chain reaction (Multiplex PCR).

**Results:**

Under optimized conditions, the *C. tropicalis*-MCDA−CRISPR/Cas12a assay was completed within approximately 53 min, with a limit of detection of 30 fg of genomic DNA per reaction. The assay showed no cross−reactivity with non−*C. tropicalis* pathogens. Clinical validation using 128 specimens demonstrated a sensitivity of 100.0% (95% CI: 93.4–100.0%) and a specificity of 97.3% (95% CI: 90.6–99.7%) against a composite reference standard, with near−perfect agreement (κ= 0.968).

**Discussion:**

The *C. tropicalis*-MCDA-CRISPR/Cas12a assay is an efficient, accurate, and practical diagnostic tool suitable for use in resource-limited laboratories.

## Introduction

Every year, 3.8 million people worldwide die from invasive fungal diseases, and approximately 70% of these deaths can be directly attributed to the fungal infection itself (Denning, 2024). Candida bloodstream infections account for 50% of such cases in Asia and over 85% of all fungal sepsis cases in Europe and the United States (Bongomin et al., 2017; Cornely et al., 2025). *Candida tropicalis* (*C. tropicalis*) is a globally distributed opportunistic pathogenic commensal yeast that can cause invasive infections at diverse sites, including the blood, heart, central nervous system, eyes, bones, and other internal organs. Invasive candidiasis has a total mortality rate of up to 55%–60% in adults and 26%–40% in children, making it the second most common *Candida* species associated with this condition (World Health Organization, 2022). A particular concern is its elevated cross-resistance rate, which has already surpassed that of the *Candida glabrata* complex and *Candida albicans* (Cornely et al., 2025). The designation of *C. tropicalis* as a critical-priority fungal pathogen in the World Health Organization fungal priority pathogen list highlights the urgency of research efforts against this pathogen (Casalini et al., 2024).

In clinical practice, up to 80% of related deaths are associated with the lack of rapid and specific diagnostic methods and effective antifungal treatment. Traditional methods to identify fungi, including Gram staining, colorimetric media, and biochemical profiling, are often complex and time-consuming. Accurate results usually rely on sophisticated equipment, including mass spectrometers or DNA sequencers, however, most routine laboratories do not have such facilities. Furthermore, the entire process requires professional operation and interpretation, and these limitations severely hinder the development of rapid and early fungal diagnosis. Therefore, establishing and implementing rapid fungal detection platforms is a critical scientific issue that urgently needs to be addressed.

Recently, significant advances have been made in non-culture-based or fungal detection technologies, including fungal antigen/antibody testing, rapid diagnostic assays, and molecular biological methods. These techniques have substantially improved the clinical diagnosis of invasive fungal infections. Among these, various nucleic acid amplification technologies, including conventional PCR (White et al., 2006), real-time fluorescent quantitative PCR, reverse transcription PCR (Bovers et al., 2007), loop-mediated isothermal amplification (LAMP) (Fallahi et al., 2020), recombinase polymerase amplification (RPA) (Koffi et al., 2025), and mass spectrometry identification (Emonet et al., 2010), have been developed and demonstrated considerable application value in detecting invasive fungi. Furthermore, these core technologies are increasingly being integrated with diverse detection platforms, including nanoparticle-based lateral flow biosensors (LFB) and restriction endonuclease-mediated fluorescence assays, to enhance their practicality and performance (Cao et al., 2022; Gu et al., 2025).

Multiple cross-displacement amplification (MCDA) is a novel isothermal amplification technique that employs ten pairs of primers targeting ten distinct regions within the target sequence (Wang et al., 2015). Owing to its simplicity, rapidity, and high sensitivity, MCDA has been widely applied to detect various pathogens, including viruses, bacteria, and others (Huang et al., 2023; Zhu et al., 2021). The amplification products of MCDA can be detected using various methods, such as agarose gel electrophoresis, lateral flow biosensors (LFB), or real-time fluorescence monitoring combined with restriction endonuclease digestion. However, these detection techniques for MCDA are often prone to generating false positive results due to non-specific amplification (Mao et al., 2025; Zhang et al., 2025). In contrast, the clustered regularly interspaced short palindromic repeats (CRISPR) and its associated proteins (Cas) system, when combined with MCDA, has formed a highly sensitive MCDA-CRISPR/Cas detection platform, effectively overcoming the aforementioned limitations. In this platform, the trans-cleavage activity of CRISPR/Cas12a is strictly dependent on precise –target DNA pairing, thereby providing a second orthogonal sequence-validation layer following MCDA amplification. Consequently, even if non-specific amplification products (e.g., primer-dimers) are generated during the MCDA stage, the Cas12a enzyme will not be activated unless the accurately recognizes the target sequence, and thus no fluorescent signal will be produced. This dual-recognition design, encompassing “amplification-layer enrichment and CRISPR-layer validation,” mechanistically suppresses false-positive signals resulting from non-specific amplification artifacts. The platform offers significant advantages, including operational simplicity, strong practicality, single-base resolution, and the capability for single-molecule detection (Sashital, 2018). Furthermore, Pathogen detection studies using CRISPR-Cas12a/Cas12b biosensing systems have consistently exhibited high sensitivity and specificity, outperforming conventional post-amplification analysis methods, including gel electrophoresis, lateral flow biosensors, or restriction enzyme analysis (Yongdong Fu et al., 2025; Chen et al., 2025; Khmeleva et al., 2024; Yang et al., 2023).

Herein, we report a novel integrated assay, designated *C. tropicalis*-MCDA-CRISPR/Cas12a, which synergizes MCDA amplification with CRISPR/Cas12a-mediated detection. This streamlined workflow was completed in 53 min, including a 13 min sample nucleic acid extraction, a 30 min MCDA step at 64 °C, and a subsequent 10 min Cas12a-based fluorescence reaction. The performance of the assay was rigorously assessed using DNA from a panel of *C. tropicalis* and related non-target strains, demonstrating high specificity. Furthermore, its clinical applicability was successfully verified using relevant samples. This method facilitates simple, rapid, and ultrasensitive detection, with results that can be interpreted by real-time fluorescence monitoring and endpoint visualization under blue light. This robust platform holds significant promise for enabling the precise and field-deployable detection of *C. tropicalis* in various contexts.

## Materials and methods

### Reagents and instruments

Nucleic acids from all clinical strains and samples were extracted using commercial kits from Tianlong Technology Co., Ltd. (Xi’an, China). The isothermal amplification reagents, including *Bst* 3.0 DNA polymerase and deoxynucleotide (dNTP) solution, were obtained from New England BioLabs (Beijing) Co., Ltd. All specific primers, crRNA, and fluorescent-quenched ssDNA reporter probes were synthesized by Beijing Tsingke Biotechnology Co., Ltd. (Beijing, China). Sabouraud dextrose agar plates (Guangzhou Dijing Co., Ltd., China) were used for fungal culture. The process involved qualitative fluorescence detection with an Applied Biosystems 7500 real-time PCR system (USA). Concurrently, endpoint visualization under blue light was achieved using an E-BOX gel documentation system (Vilber Bio-Imaging, France). Other equipment used included a YCP-series CO_2_ incubator (Changsha Huaxi Electronic Technology, China), a Qsep400 Fully Automated Nucleic Acid and Protein Analysis System (BioHelix Corporation, China), and an Autof TCX Automated Microbial Identification System (Autobio Diagnostics, China).

### Primer and crRNA design

The MCDA primers used in this study were designed following a previously established method targeting the *C. tropicalis-*specific ITS II gene (Accession No. AF268095.1) using Primer Explorer V4 and Primer software (version 5.0) (Wang et al., 2015, Wang et al., 2021). Based on the detection principle of the CRISPR/Cas12a system, we made key modifications to the CP1 primer. At the junction of the C1 primer and the P1 primer, a specific PAM site (5’-TTTV-3’) recognized by the Cas12a effector protein was added (where V represents A/C/G). Since “TTTC” (V = C) is one of the PAM combinations with the highest cutting efficiency of this protein in human cells and *in vitro* systems, we chose 5’-TTTC-3’ for the primer modification in this experiment (Zetsche et al., 2015; Gao et al., 2016; Kleinstiver et al., 2019). In this design, the crRNA serves as a perfect antisense strand to the P1 primer sequence, allowing the CRISPR/Cas12a system to directly recognize and respond to the presence of the primer in the amplification products. Furthermore, an ssDNA reporter probe was synthesized, with a FAM fluorophore at the 5’ end and a BHQ1 quencher at the 3’ end. All oligonucleotide sequences used in this study are detailed in **Supplementary Table 1**, **Supplementary Figure 1**.

### Bacterial strain and clinical samples

The standard strains of *C. tropicalis* used in this study were sourced from inter-laboratory quality assessment programs. Specifically, three *C. tropicalis* strains from the annual microbiome quality assessment panels (2022-2024) were selected for MCDA primer validation to determine the optimal strain for constructing the experimental system. The experimental results of this selection process are presented in **Supplementary Figure 2**. The strain panel comprised 12 C*. tropicalis* isolates (four EQA program strains, including three aforementioned isolates and reference strain 20253008 from the Guizhou 2025 EQA program, plus eight clinically validated isolates confirmed by MALDI-TOF MS), 22 non-*C. tropicalis Candida* species, and five viral or atypical pathogen strains. All strains were obtained from the National or Guizhou Provincial Quality Control Programs, Ministry of Health Reference Collections, or were clinically derived isolates confirmed by mass spectrometry. The viral and atypical pathogen strains used were pre-verified using commercial nucleic acid amplification kits. Additionally, 128 samples from patients with suspected *C. tropicalis* infections were collected from *the First People’s Hospital of Guiyang* for clinical validation (The isolates were derived from multiple clinical sources, encompassing a broad spectrum of specimen types such as blood, urine, pus, pleural/peritoneal fluid, bronchoalveolar lavage fluid, and sputum). Nucleic acids from all pure cultures and clinical specimens were extracted and purified using a commercial genomic DNA kit. Isothermal amplification was performed at 64 °C for 30 min (Wang Y et al., 2021). The study protocol was approved by the ethics committee of *the First People’s Hospital of Guiyang*.

### *C. tropicalis*-MCDA pre-amplification reaction

The standard MCDA reaction was performed in a 25 µL mixture containing the following components: 0.4 µM each of primers F1 and F2, 0.8 µM each of primers C1, C2, D1, D2, R1, and R2, 1.6 µM each of primers CP1 and CP2, 2.5 µL of 10× isothermal amplification buffer II, 1.5 µL of 6 mM MgSO_4_, 3.5 µL of 1.4 mM dNTP mix, 1 µL of *Bst* 3.0 DNA polymerase (8 U), and 1 µL (for pure cultures) or 5 µL (for clinical samples) of the DNA template. Nuclease-free water was added to obtain a final volume of 25 µL. The isothermal amplification was conducted at 64 °C for 30 min. For system optimization, the reference strain used was *C. tropicalis* (strain number: 202408), obtained from the second round of the 2024 microbial inter-laboratory quality assessment organized by the National Centre for Clinical Laboratories. Genomic DNA from *Candida albicans* ATCC 10231 and *Escherichia coli* ATCC 25922 served as negative controls, with nuclease-free water used as a blank control.

### *C. tropicalis*-MCDA-CRISPR/Cas12a trans-cleavage assay

The pre-amplified MCDA products were detected using the CRISPR/Cas12a biosensing system. First, the CRISPR/Cas12a-crRNA complex was prepared by mixing 10 μL of Cas12a enzyme (1 μM) with 10 μL of crRNA (1 μM) in 80 μL of 2×NE buffer, resulting in a total volume of 100 μL. The complex was then incubated at 37 °C for 10 minutes. The complex can be used immediately or stored at 0–4 °C for no more than 12 hours before use (Jia et al., 2023). Subsequently, the CRISPR/Cas12a trans-cleavage reaction was performed in a 50 µL mixture containing 9 µL of the pre-assembled CRISPR/Cas12a–crRNA complex, 1 µL of MCDA amplification products, 1.25 µL of single-stranded DNA (ssDNA) reporter probe, 25 µL of 2× NEBuffer, and 13.75 µL of nuclease-free water. The reaction mixture was incubated in a real-time fluorescence PCR instrument at 36–40 °C for 5–20 minutes, during which fluorescence signals were monitored in real time. In addition, endpoint results could be visualized under blue light illumination.

To prevent aerosol contamination, the entire experimental workflow was conducted in four physically separated areas: reagent preparation, nucleic acid extraction, MCDA amplification, and CRISPR/Cas12a detection. A unidirectional workflow was strictly enforced, and filter-barrier pipette tips were used throughout all steps. Sterile nuclease-free water was included as a negative control in each run to monitor potential cross-contamination. All manipulation steps were performed in a Class II biological safety cabinet.

### Optimization of CRISPR/Cas12a‐mediated reaction

To determine the optimal reaction conditions for the *C. tropicalis* MCDA-CRISPR/Cas12a trans-cleavage assay, several key parameters were systematically screened and optimized. First, the reaction temperature was optimized by testing five different temperatures (36, 37, 38, 39, and 40 °C) with a fixed reaction time of 10 minutes. Second, the reaction time was evaluated at 5-minute intervals (5, 10, 15, and 20 minutes) under the optimal temperature. Third, the volume of MCDA amplification products added to the CRISPR/Cas12a reaction was titrated from 1 µL to 5 µL at 1-µL increments to determine the optimal input amount. Fourth, the concentration of the ssDNA reporter probe was optimized across a range of concentrations (10, 20, 50, and 100 µM). Fifth, each individual component of the reaction system (probe, Cas12a, crRNA, buffer, and nuclease-free water) was systematically evaluated to verify its necessity and contribution to the overall reaction. Finally, a checkerboard titration was performed to determine the optimal concentration ratio of the Cas12a–crRNA complex, in which five concentrations of Cas12a (10, 1, 0.5, 0.2, and 0.1 µM) and five concentrations of crRNA (10, 1, 0.5, 0.2, and 0.1 µM) were cross-tested in a 5 × 5 matrix, yielding 25 independent combinations. The combination yielding the highest fluorescence signal-to-background ratio was selected as the optimal formulation for subsequent experiments.

### Sensitivity evaluation of *C. tropicalis*-MCDA-CRISPR/Cas12a assay

The standard strain was inoculated onto Sabouraud dextrose agar and incubated at 35 °C for 24 h. Genomic DNA was extracted from the pure culture, and its concentration was measured in triplicate, confirming an initial concentration of 30 ng/µL. This initial extract was then subjected to a ten-fold serial dilution series, ranging from 30 ng/µL to 3 fg/µL. These diluted samples were used to determine the limit of detection (LoD) of the assay. Each dilution level was analyzed in triplicate.

### Specificity evaluation of *C. tropicalis*-MCDA-CRISPR/Cas12a assay

The specificity of the assay was evaluated using 12 C*. tropicalis* strains and 27 non-*C. tropicalis Candida* strains (including unicellular fungi, multicellular fungi, bacteria, viruses, and atypical pathogens. For details, please refer to **Supplementary Table 2**.) Nuclease-free water served as the negative control. All reactions were performed under optimized conditions and repeated in triplicate.

### Repeatability evaluation of the *C. tropicalis*-MCDA-CRISPR/Cas12a assay

To evaluate assay repeatability, nine *C. tropicalis*-positive specimens (genomic DNA extracted from clinically identified pure cultures) and three non-*C. tropicalis Candida* specimens were selected for intra- and inter-assay testing. Each sample was analyzed in triplicate. The consistency rate was calculated as follows: (number of concordant results/total number of tests) × 100%.

### Clinical sample testing

To perform clinical validation and methodological comparison of the *C. tropicalis*-MCDA-CRISPR/Cas12a assay, this study enrolled 128 clinical specimens (blood, urine, pus, pleural/peritoneal fluid, bronchoalveolar lavage fluid, and sputum). These specimens were tested in parallel by conventional culture, multiplex PCR combined with capillary electrophoresis, and the *C. tropicalis*-MCDA-CRISPR/Cas12a assay. Specimens were randomly collected, and all assays were performed independently by different operators in a blinded manner, with results compared only after all tests were completed.

### Experimental data analysis

Raw amplification data were collated using WPS Office Excel. The experimental flowchart was completed using . All statistical analyses were performed using GraphPad Prism software (version 10.5.0; GraphPad Software, San Diego, CA, USA). Statistical differences between groups were determined by comparison to the control group. Data are presented as the mean from three independent replicates (n = 3). Significance levels are denoted as follows: ***P < 0.001, **P < 0.01, *P < 0.05; “ns” indicates a non-significant difference.

## Results

### Detection principle of *C. tropicalis*-MCDA-CRISPR/Cas12a assay

**Figure 1:**
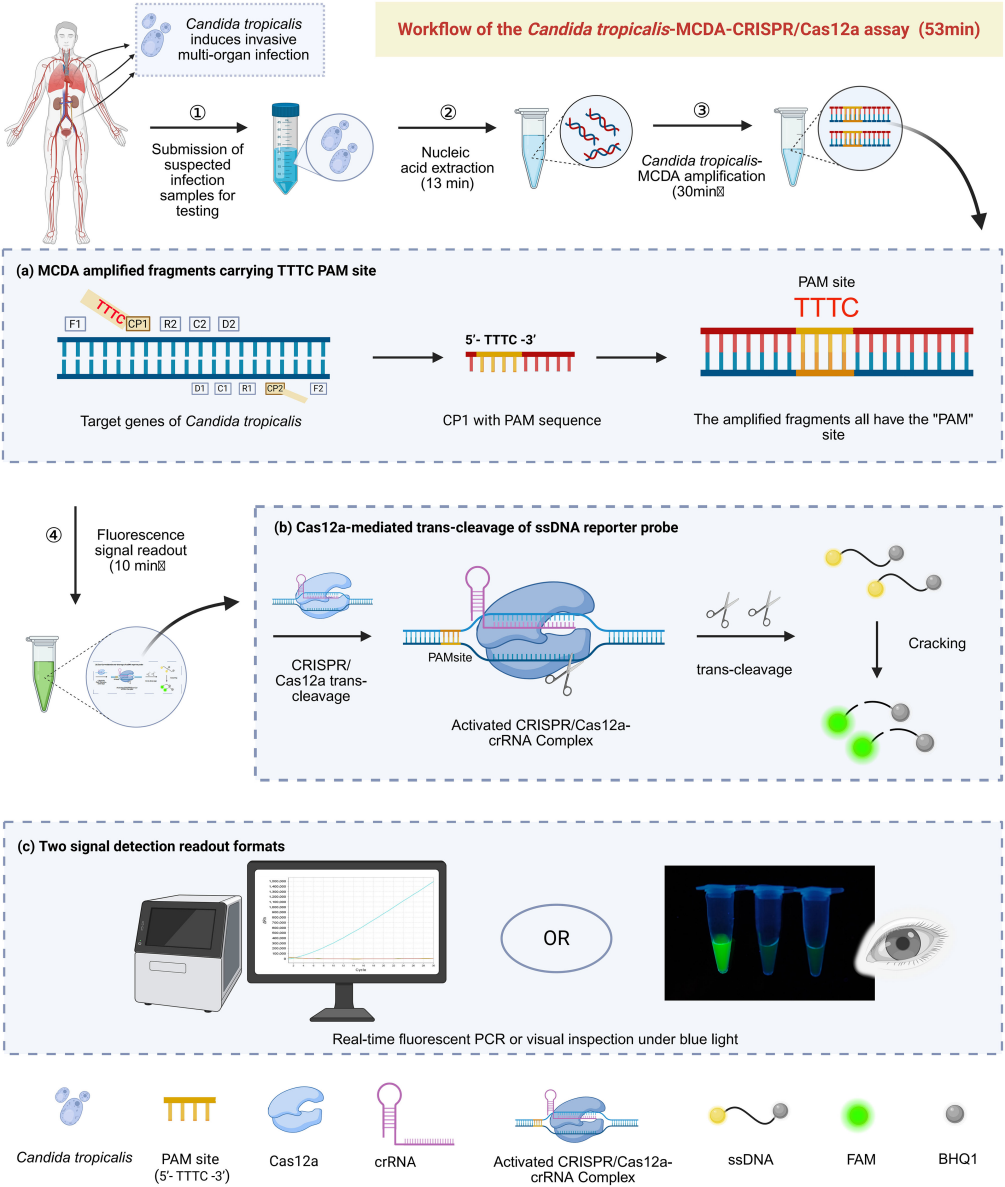
Principle and workflow of the *C. tropicalis*-MCDA-CRISPR/Cas12a assay. **(a)** MCDA amplification of target genes, with the cross primer CP1 incorporating a PAM sequence (5′-TTTC-3′). **(b)** Cas12a–crRNA complex specifically recognizes the PAM-containing amplicons, activating trans-cleavage of the ssDNA reporter probe. **(c)** Two signal readout formats: real-time fluorescence detection and visual inspection under blue light. Workflow steps ①–④: clinical specimen receipt → DNA extraction (13 min) → MCDA amplification (30 min) → CRISPR/Cas12a detection (10 min), with a total turnaround time of ~53 min. Created in BioRender. Ye, R. (2026) https://BioRender.com/7aq2h24.

The detection principle and workflow of the *C. tropicalis* MCDA-CRISPR/Cas12a assay are illustrated in **Figures 1a-c** and **Figure 1** (①–④), respectively. In the MCDA reaction, the cross primer CP1 was specifically designed to contain the protospacer-adjacent motif (PAM) sequence (5′-TTTC-3′) required for Cas12a recognition. Upon completion of amplification, the resulting amplicons can be specifically recognized by the Cas12a–crRNA complex, in which the crRNA is designed to be complementary to the P1 primer region incorporated within the amplified products. Upon target binding, the trans-cleavage activity of Cas12a is activated, leading to non-specific cleavage of the dual-labeled single-stranded DNA reporter probe (5′-FAM and 3′-BHQ1), thereby generating a fluorescent signal. This signal can be monitored in real time using a real-time fluorescence PCR instrument or visually inspected at the endpoint under blue light illumination.

The entire detection workflow is as follows: upon receipt of clinical specimens from patients with suspected invasive C. tropicalis infection, the procedure sequentially comprises DNA extraction (13 min), MCDA pre-amplification (30 min), and CRISPR/Cas12a trans-cleavage detection (10 min). The whole process can be completed within approximately 53 minutes from sample to result.

### Optimization of the reaction conditions for *C. tropicalis*-MCDA-CRISPR/Cas12a assay

**Figure 2:**
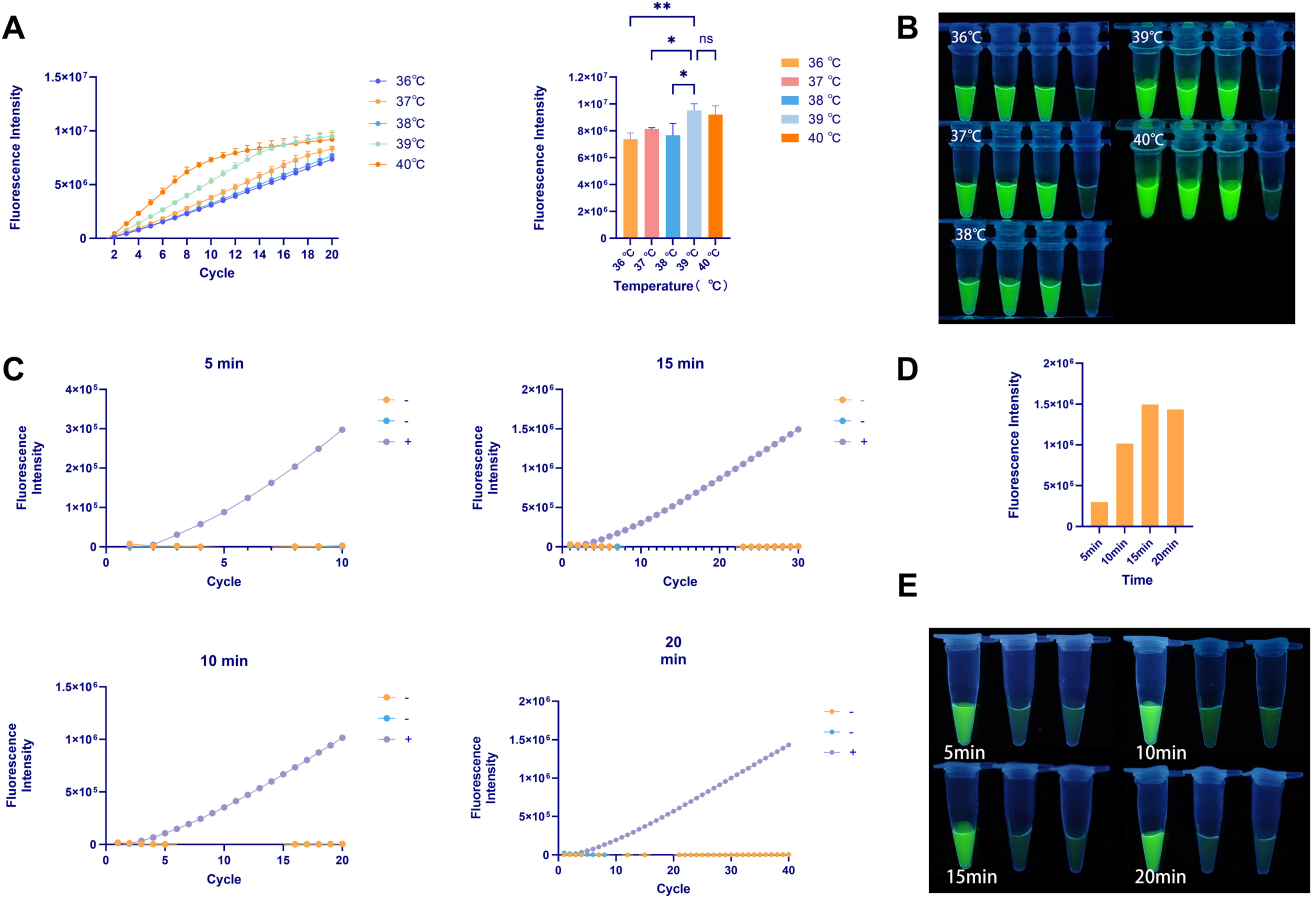
Optimization of the reaction temperature and time for the *C. tropicali*s-MCDA-CRISPR/Cas12a assay. **(A, B)** Fluorescence intensity at different temperatures (36–40 °C) presented as bar charts with corresponding blue-light visual results. The highest intensity was observed at 39 °C, significantly higher than at 36–38 °C, but comparable to 40 °C. **(C–E)** Fluorescence intensity at different reaction times (5–20 min). A marked increase was observed within 10 min. *C. tropicalis* reference strain (No. 202408) served as a positive control, and nuclease-free water as a blank control. Data are shown as mean ± SD (n = 3). Statistical significance was determined by one-way ANOVA followed by Tukey’s multiple comparison test. **P < 0.01, *P < 0.05; “ns” indicates a non-significant difference.

To establish the optimal reaction conditions for the CRISPR/Cas12a detection system, the reaction temperature and time were first systematically evaluated. A temperature gradient ranging from 36 to 40 °C (in 1 °C increments) was tested with a fixed reaction time of 10 min. The results showed that 39 °C yielded the highest fluorescence signal and was therefore selected as the optimal temperature for all subsequent experiments (**Figures 2A, B**). Next, the reaction time was assessed at 39 °C over a time course of 5, 10, 15, and 20 min, revealing that a significant fluorescent signal was generated within 10 min (**Figures 2C–E**). Accordingly, the optimal reaction conditions for the *C. tropicalis*–MCDA–CRISPR/Cas12a assay were determined as 39 °C for 10 min.

**Figure 3:**
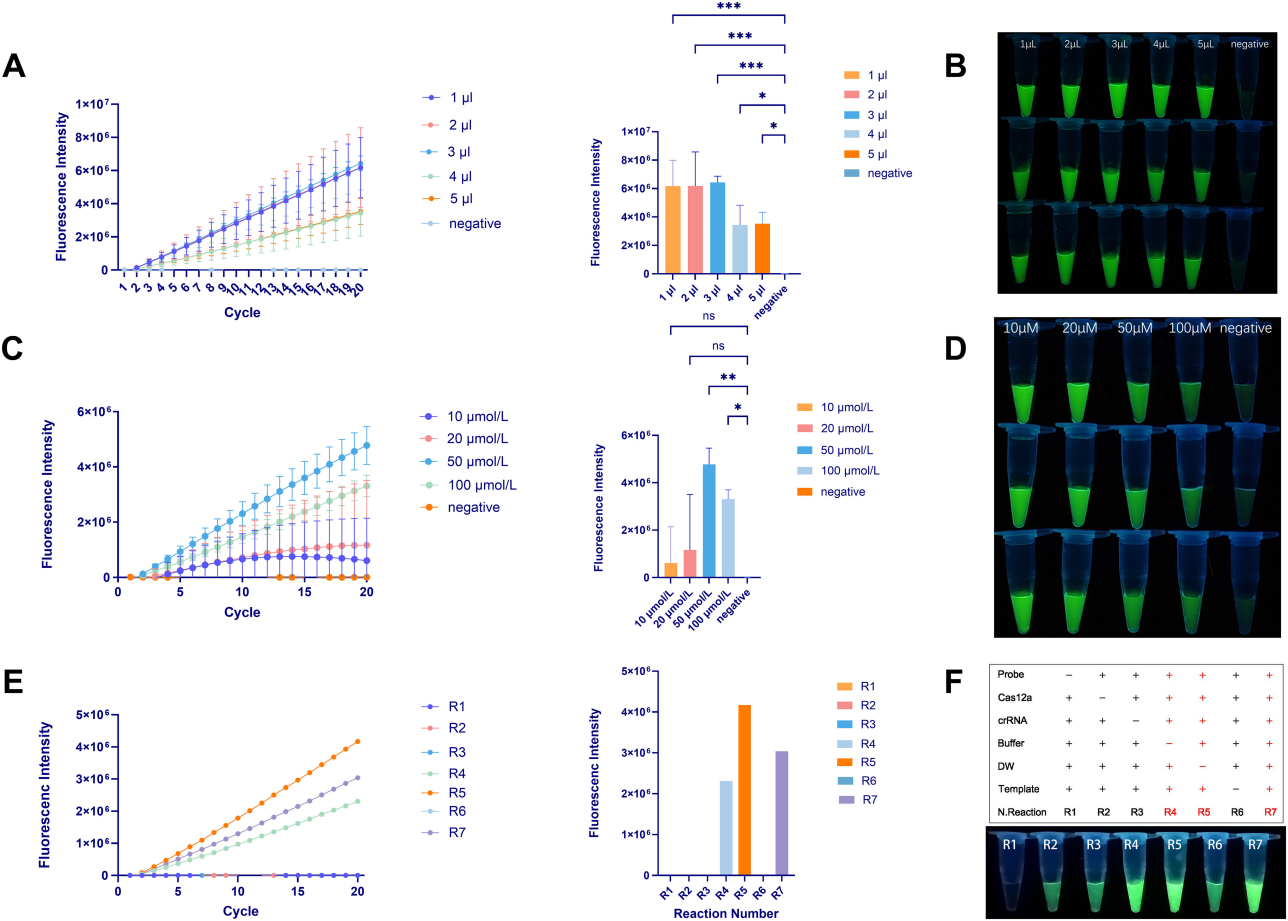
Optimization of template volume and probe concentration, and validation of the reaction system components for the *C. tropicali*s-MCDA-CRISPR/Cas12a assay. **(A, B)** Fluorescence intensity at different MCDA product input volumes (1–5 µL) presented as bar charts with corresponding blue-light visual results. All volumes from 1 µL to 3 µL showed significantly higher fluorescence than the negative control, with the highest intensity observed at 3 µL. **(C, D)** Fluorescence intensity at different ssDNA reporter probe concentrations (10–100 µmol/L) presented as bar charts with corresponding blue-light visual results. The 50 µmol/L concentration showed the most significant difference compared with the negative control. **(E, F)** Verification of the necessity of each reaction component, presented as bar charts. Significant fluorescent signals were observed in R4, R5, and R7, indicating that the probe, Cas12a protein, crRNA, and template are all essential for a successful reaction. *C. tropicalis* reference strain (No. 202408) served as the positive control, and nuclease-free water served as the blank control. All experiments were performed independently in triplicate. Data are shown as mean ± SD (n = 3). Statistical significance was determined by one-way ANOVA followed by Tukey’s multiple comparison test. ***P < 0.001, **P < 0.01, *P < 0.05; “ns” indicates a non-significant difference.

**Figure 4:**
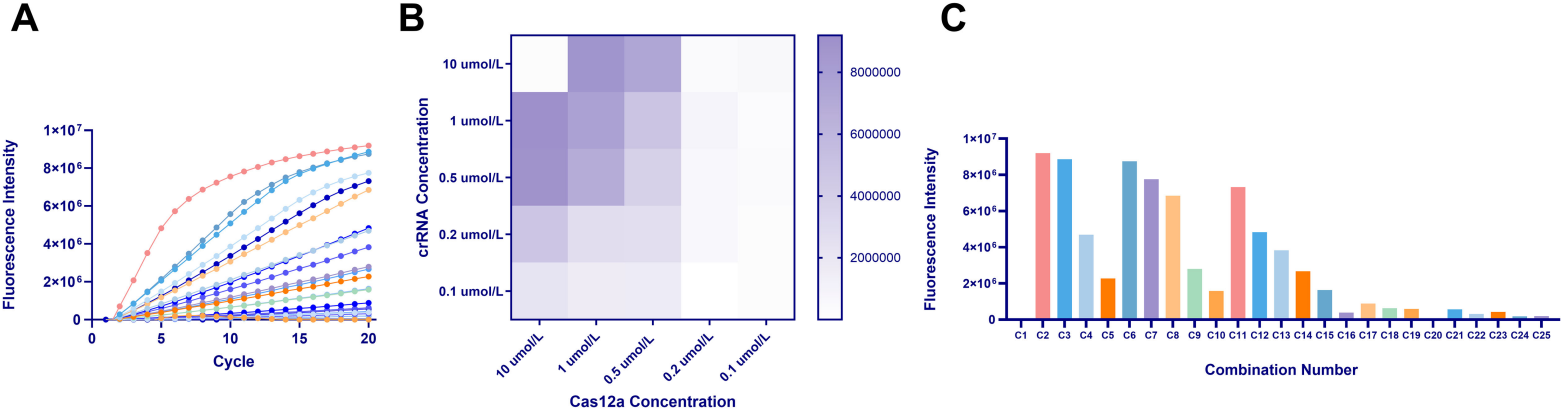
Optimize the concentration ratio of crRNA and Cas12a complex through a grid-based experiment. **(A)** Amplification curves of 25 Cas12a–crRNA concentration combinations. **(B)** Corresponding heatmap of fluorescence intensities. **(C)** Bar chart comparing fluorescence intensities of each combination. A 5 × 5 checkerboard titration was performed with five Cas12a concentrations (10, 1, 0.5, 0.2, and 0.1 µM) and five crRNA concentrations (10, 1, 0.5, 0.2, and 0.1 µM). The combination of 10 µM Cas12a with 1 µM crRNA (C2) exhibited the highest fluorescence signal and was adopted as the final formulation.

To further enhance the trans-cleavage efficiency of Cas12a, several additional parameters were optimized: the input volume of pre-amplified MCDA products, the concentration of the single-stranded DNA (ssDNA) reporter probe, the necessity of each reaction component, and the optimal concentration ratio of the Cas12a–crRNA complex. The optimal input volume was determined to be 3 µL (**Figures 3A, B**), and the optimal ssDNA reporter probe concentration was 50 µM (**Figures 3C, D**). Verification of the necessity of each component confirmed that, aside from the universal buffer and nuclease-free water, the probe, Cas12a protein, crRNA, and template were all essential for a successful reaction (**Figures 3E, F**). Finally, a 5 × 5 checkerboard titration was performed to determine the optimal Cas12a–crRNA complex concentration ratio, with five concentrations of Cas12a (10, 1, 0.5, 0.2, and 0.1 µM) and five concentrations of crRNA (10, 1, 0.5, 0.2, and 0.1 µM) tested in 25 independent combinations. The combination of 10 µM Cas12a with 1 µM crRNA (designated as combination C2) exhibited the highest fluorescence signal and was therefore adopted as the final formulation. All optimization experiments were performed in triplicate (**Figure 4**).

### Sensitivity evaluation of *C. tropicalis*-MCDA-CRISPR/Cas12a assay

**Figure 5:**
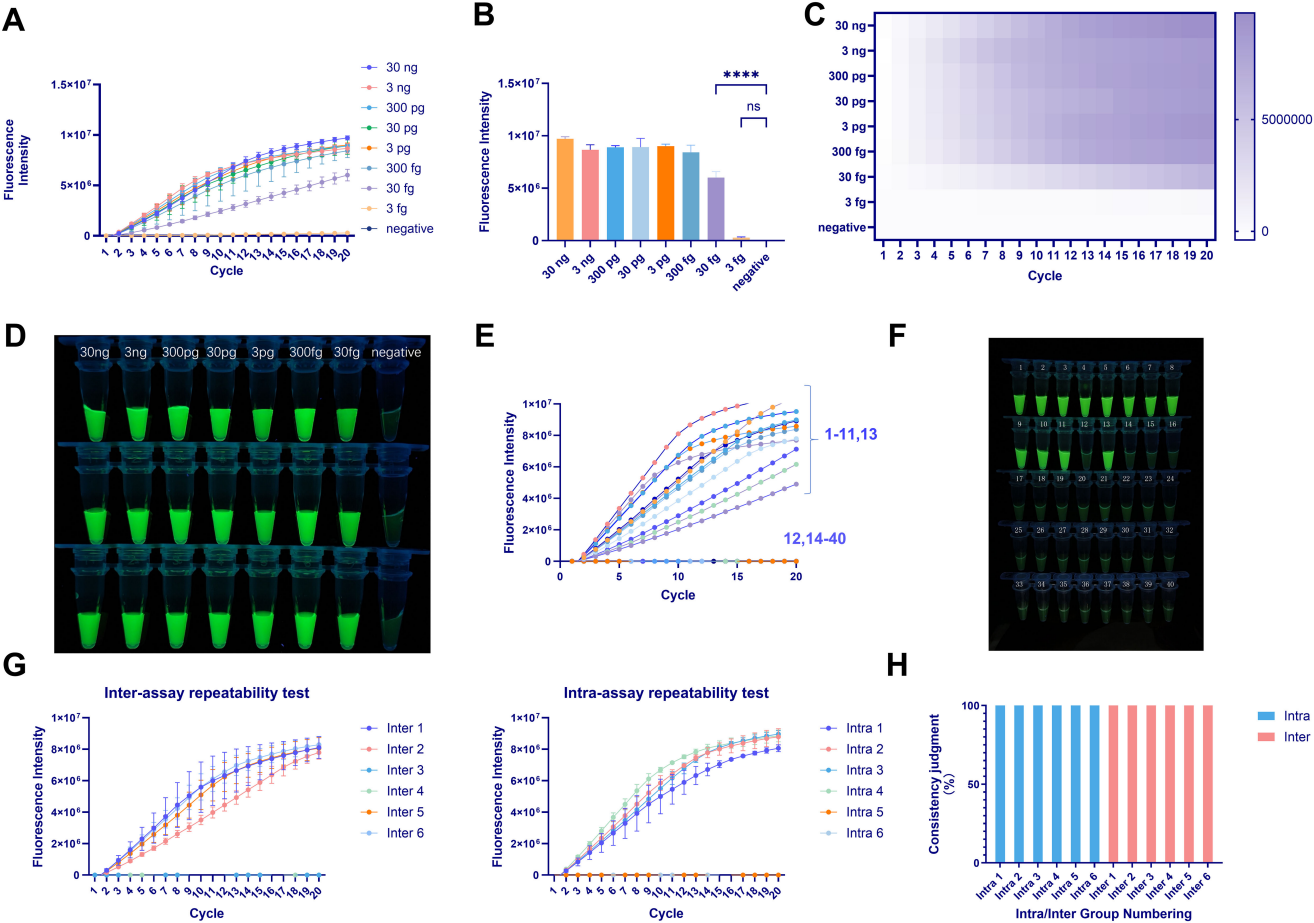
Sensitivity, specificity and repeatability evaluation of the *C. tropicali*s-MCDA-CRISPR/Cas12a assay. **(A–D)** Sensitivity evaluation using serially diluted *C. tropicalis* genomic DNA (30 ng/μL to 3 fg/μL). Fluorescence intensities were presented as bar charts and heatmaps, with corresponding blue-light visual results. The LoD (30 fg per reaction) showed significantly higher fluorescence than 3 fg per reaction and the negative control. **(E, F)** Specificity evaluation using 12 C*. tropicalis* strains and 27 non-*C. tropicalis* strains, presented as amplification curves with corresponding blue-light visual results. No cross-reactivity was observed. **(G, H)** Intra-assay and inter-assay repeatability evaluation, presented as real-time amplification curves and concordance analysis. Data are shown as mean ± SD (n = 3). Statistical significance was determined by one-way ANOVA followed by Tukey’s multiple comparison test. ****P < 0.0001, indicates a non-significant difference.

The sensitivity of the *C. tropicalis*-MCDA-CRISPR/Cas12a assay was evaluated using serially diluted genomic DNA extracted from a standard strain. The initial DNA concentration of 30 ng/µL was subjected to ten-fold serial dilutions, yielding a concentration series from 30 ng/µL to 3 fg/µL (30 ng/µL, 3 ng/µL, 300 pg/µL, 30 pg/µL, 3 pg/µL, 300 fg/µL, 30 fg/µL, and 3 fg/µL). Each dilution was tested in three independent replicates. The results demonstrated that the assay could consistently detect the target down to a concentration of 30 fg per reaction, establishing this as the LoD (**Figures 5A–D**).

### Specificity evaluation of *C. tropicalis*-MCDA-CRISPR/Cas12a assay

For specificity assessment, the assay was challenged with a panel of 12 C*. tropicalis* strains and 27 non-*C. tropicalis Candida* strains. Positive results were exclusively observed in reactions containing *C. tropicalis* genomic DNA, while all reactions with non-target *Candida* species yielded negative results, confirming 100% analytical specificity for *C. tropicalis* (**Figures 5E, F**).

### Repeatability evaluation of *C. tropicalis*-MCDA-CRISPR/Cas12a assay

The repeatability of the assay was determined through intra- and interassay testing. This study involved nine *C. tropicalis*-positive clinical isolates and three non-*C. tropicalis* controls, with each sample analyzed in three separate replicates. Both intra- and inter-assay tests achieved 100% consistency, indicating high reliability and stability of the established method. The repeatability verification and consistency results are summarized in **Supplementary Table 3** and detailed in **Figures 5G, H**, respectively.

### Clinical sample validation of *C. tropicalis*-MCDA-CRISPR/Cas12a assay

A composite reference standard (CRS) was established to resolve discrepancies among the three methods: a sample was defined as CRS-positive if either culture or multiplex PCR yielded a positive result, and CRS-negative only when both methods yielded negative results. This approach was adopted to compensate for the limited sensitivity of any single reference method (Alonzo and Pepe, 1999; Schiller et al., 2016). Pairwise agreement analyses were also performed between the *C. tropicalis*-MCDA-CRISPR/Cas12a assay and the two comparator methods: the assay showed 96.1% (123/128) agreement with fungal culture and 98.4% (126/128) agreement with multiplex PCR.

**Figure 6:**
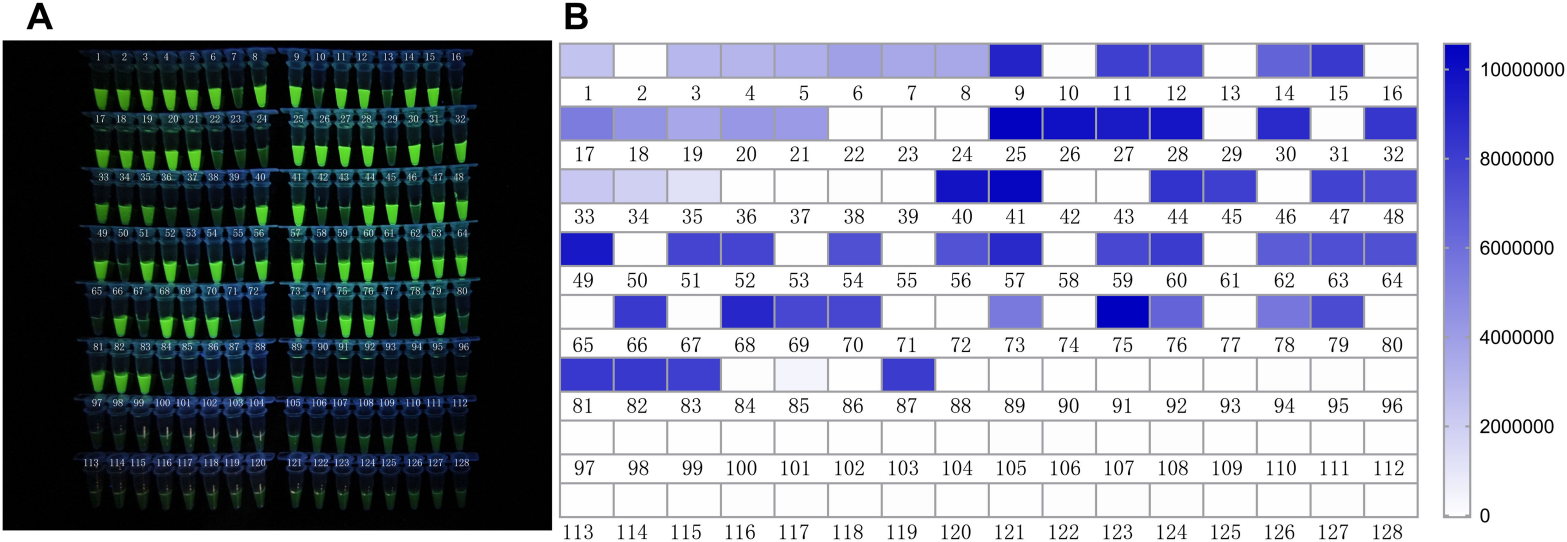
Clinical validation of the *C. tropicali*s-MCDA-CRISPR/Cas12a assay. **(A)** Visual inspection results of all 128 clinical specimens under blue light, arranged in a 16 × 8 layout. Positive samples showed obvious fluorescence. **(B)** Heatmap of fluorescence intensity for each specimen, with dark blue representing the highest fluorescence intensity and white representing the lowest. Results were compared with the composite reference standard.

Among the 128 clinical specimens, the *C. tropicalis*-MCDA-CRISPR/Cas12a assay identified 56 positive cases (**Figures 6A, B**), compared with 51 by culture and 54 by multiplex PCR. Using the CRS as the reference, the assay correctly identified 54 true positives and 72 true negatives, with 2 false positives (**Table 1**). The corresponding diagnostic performance metrics are summarized in **Table 2**: the assay demonstrated a sensitivity of 100.0% (95% CI: 93.4–100.0%), a specificity of 97.3% (95% CI: 90.6–99.7%), a positive predictive value of 96.4% (95% CI: 87.5–99.6%), a negative predictive value of 100.0% (95% CI: 95.0–100.0%), and an accuracy of 98.4% (95% CI: 94.4–99.8%). The Cohen’s kappa coefficient between the assay and the CRS was 0.968 (95% CI: 0.923–1.000), indicating near-perfect agreement. Detailed cross-tabulations of all three methods are provided in **Supplementary Tables 4**, **5**.

**Table 1:** Diagnostic performance and pairwise agreement of the *C. tropicalis*-MCDA-CRISPR/Cas12a assay based on the composite reference standard (CRS).

The *C. tropicalis*-MCDA-CRISPR/Cas12a assay	CRS positive (n = 54)	CRS negative (n = 74)	Total	Concordance with fungal culture	Concordance with multiplex PCR
Assay positive	54 (TP)	2 (FP)	56	96.1% (123/128)	98.4% (126/128)
Assay negative	0 (FN)	72 (TN)	72		
Total	54	74	128		

TP, true positive; FP, false positive; FN, false negative; TN, true negative. CRS positive was defined as positive by either fungal culture or multiplex PCR; CRS negative was defined as negative by both methods. The two false-positive cases (MCDA-positive but CRS-negative) were negative by both culture and multiplex PCR, potentially reflecting true infections with non-viable organisms or low fungal burden, or possible cross-reactivity.

**Table 2:** Diagnostic performance metrics of the *C. tropicalis*-MCDA-CRISPR/Cas12a assay (n = 128).

Metric	Value	95% Confidence interval
Sensitivity	100.0%	93.4% – 100.0%
Specificity	97.3%	90.6% – 99.7%
Positive Predictive Value (PPV)	96.4%	87.5% – 99.6%
Negative Predictive Value (NPV)	100.0%	95.0% – 100.0%
Overall Accuracy	98.4%	94.4% – 99.8%
Cohen’s Kappa Coefficient	0.968	0.923 – 1.000

CRS was defined as positive if either culture or multiplex PCR was positive, and negative only when both were negative. CI, confidence interval.

Of note, the two specimens that were positive by the MCDA-CRISPR/Cas12a assay but negative by both culture and multiplex PCR (i.e., false positives relative to the CRS) were identified. Although Sanger sequencing was not performed in the present study due to its high cost, long turnaround time (3–5 days), and low throughput, we acknowledge that sequencing would be required in future studies to definitively resolve such discrepancies. Despite this limitation, the high sensitivity (100%) and near-perfect agreement with the CRS (κ = 0.968) suggest that the *C. tropicalis*-MCDA-CRISPR/Cas12a assay may offer enhanced detection capability compared with culture and multiplex PCR. The assay therefore holds significant promise as a rapid and accurate molecular diagnostic tool for the detection of *C. tropicalis* infections in clinical settings. Future multicenter prospective studies incorporating sequencing confirmation are warranted to further validate these findings.

## Discussion

Delays in diagnosing fungal infections have hindered effective early intervention and clinical management of invasive fungal diseases, contributing to increased healthcare resource utilization, economic burden, and mortality. In invasive candidiasis, more than half of the isolated Candida strains are non-albicans species, among which *Candida tropicalis* is the most prevalent (22.74%), followed by *Candida glabrata* (17.50%) and *Candida parapsilosis* (11.02%). As the second most common causative agent of Candida infections, *C. tropicalis* holds significant clinical importance in cases of invasive non-albicans candidiasis (Xiao et al., 2018; Ji et al., 2024). Accordingly, research on improved diagnostic techniques for this pathogen is of considerable applied value and is expected to provide an effective basis for the early diagnosis of invasive candidiasis.

Among various isothermal amplification techniques, MCDA offers several distinctive advantages for clinical pathogen detection. Compared with LAMP, MCDA employs a 10-primer system that significantly improves reaction specificity and reduces the risk of non-specific amplification (Notomi et al., 2000; Yao et al., 2024; Song et al., 2024). Compared with RPA, MCDA does not require expensive and temperature-sensitive multi-enzyme complexes, making it more cost-effective and robust for resource-limited settings (Piepenburg et al., 2006; Liu et al., 2023; Lei Wang et al., 2022). Although MCDA-LFB has been previously reported for *C. tropicalis* detection (Wang et al., 2024), our study integrates MCDA with CRISPR/Cas12a trans-cleavage activity for the first time, adding an extra layer of sequence-specific confirmation that enhances diagnostic reliability. A systematic comparison of these isothermal amplification methods is provided in **Supplementary Table 6**. The CRISPR/Cas system has been widely applied in gene editing and molecular diagnostics (Pandey et al., 2025). As a key effector of the class II type V system, Cas12a is an RNA-guided DNA endonuclease that exhibits trans-cleavage activity upon target recognition via crRNA, enabling non-specific cleavage of nearby ssDNA molecules (Paul and Montoya, 2020; Chen et al., 2018; Nguyen et al., 2024; Martin Pacesa et al., 2024). Leveraging this property, the CRISPR/Cas12a system can be engineered to detect pathogenic nucleic acids by designing a crRNA complementary to the target fragment containing a specific PAM. In previous studies, the ITS2 region has been the most frequently used target for molecular detection of *C. tropicalis* (Talukdar et al., 2021; Wang et al., 2024; Yuan et al., 2026). Accordingly, we employed the ITS2 region to design specific crRNA and combined it with MCDA pre-amplification to establish the *C. tropicalis*-MCDA-CRISPR/Cas12a assay.

Following systematic optimization of the reaction conditions, the assay produced a distinct fluorescent signal after a 10 min reaction at 39 °C, and the results were also visible under blue light illumination without requiring specialized instrumentation. The analytical sensitivity of the *C. tropicalis*-MCDA-CRISPR/Cas12a assay reached a limit of detection (LoD) of 30 fg per reaction, which is comparable to that of other recently developed bacterial detection platforms (Jia et al., 2025; Liu et al., 2025). Specificity testing using 12 C*. tropicalis* strains and 27 non-*C. tropicalis* strains demonstrated no cross-reactivity, yielding 100% specificity. The repeatability of the assay was confirmed by intra- and inter-assay testing, with 100% concordance across all replicates. The clinical performance of the assay was further evaluated using 128 clinical specimens. Among these, 56 were positive and 72 were negative by the *C. tropicalis*-MCDA-CRISPR/Cas12a assay, compared with 51 positives by fungal culture and 54 positives by multiplex PCR. Using a composite reference standard, the assay demonstrated a sensitivity of 100.0% (95% CI: 93.4–100.0%), a specificity of 97.3% (95% CI: 90.6–99.7%), and near-perfect agreement with the reference assay (κ = 0.968). These findings indicate that the established assay may offer enhanced detection capability compared with culture and multiplex PCR, and holds promise as a rapid and accurate molecular diagnostic tool for the detection of *C. tropicalis* infections in clinical settings. The entire detection process could be completed within approximately 53 min, which included nucleic acid extraction (13 min), MCDA pre-amplification (30 min), and CRISPR/Cas12a trans-cleavage detection (10 min). The detection results can be read out using a real-time fluorescence PCR instrument or visualized under blue light, a feature that makes this method more suitable for resource-limited settings, while overcoming the limitations of visual interpretation in LFB and avoiding the high costs associated with restriction enzyme recognition. However, this method has several limitations.

First, the assay currently provides only a qualitative readout and cannot quantify the pathogen load in clinical samples. In this study, the limit of detection was determined to be 30 fg per reaction (approximately equivalent to 1–2 cells/μl based on theoretical estimation). However, accurate quantification remains challenging due to the lack of a fixed conversion factor between DNA concentration and CFU counts. Therefore, developing a quantitative detection platform remains an important direction for future research (Vasconcelos et al., 2014; Thaweboon and Thaweboon, 2020; Ghafari et al., 2024). Second, sequencing was not performed to resolve the two discordant cases that were positive only by the MCDA-CRISPR assay. Consequently, we cannot definitively conclude that these represent true positives, and the possibility of false-positive results due to cross-reactivity cannot be entirely excluded. Future studies incorporating sequencing as an independent reference are warranted to further validate the specificity of the MCDA-CRISPR assay. Third, although the qualitative results were highly consistent, fluorescence intensity varied across replicate experiments, indicating the need for more robust signal standardization. Additionally, the two-step workflow requires tube opening and product transfer, which increases the risk of aerosol contamination. Nonetheless, the assay’s dual-recognition mechanism, which combines MCDA amplification with CRISPR/Cas12a-mediated validation, helps suppress non-specific signals, and strict contamination controls including four-zone separation, a unidirectional workflow, and filter tips were implemented during testing. We acknowledge this limitation and are currently exploring a one-pot system to eliminate the transfer step in future versions. Finally, The current detection methods can only detect *C. tropicalis*. Given that mixed Candida infections commonly occur in clinical settings, a multiplex detection platform capable of simultaneously identifying multiple species (such as *Candida albicans*, *Candida tropical*, and *Candida glabrata* species) would have broader clinical utility. The MCDA-CRISPR/Cas12a platform established in this study can readily achieve multiplexing by introducing species-specific crRNAs. To overcome the limitations of the current two-step workflow and expand the detection spectrum, future efforts will focus on two directions: first, developing a one-step CRISPR-based detection system for *C. tropicalis* to minimize contamination risks and improve operational efficiency, and second, adapting the MCDA-CRISPR/Cas12a platform for multiplex detection by incorporating species-specifics, enabling simultaneous identification of multiple Candida species. Additionally, future multicenter prospective studies with larger sample sizes and sequencing confirmation are warranted to further validate these findings.

In summary, the entire detection process can be completed within 53 min, including 13 min for genomic DNA extraction, 30 min for MCDA-based amplification, and 10 min for fluorescence signal detection. In resource-limited settings, the results can be visualized under blue light illumination without the need for sophisticated instrumentation. Owing to its high sensitivity, specificity, and reproducibility, together with its simplicity, rapidity, and minimal equipment requirements, the assay demonstrates strong potential for clinical diagnosis of *C. tropicalis* infections and is particularly suitable for deployment in resource-limited laboratory environments.

## Conclusion

In this study, we successfully established an MCDA-CRISPR/Cas12a assay for the detection of *C. tropicalis*. The assay achieved a limit of detection of 30 fg of genomic DNA per reaction and demonstrated excellent specificity, with no cross-reactivity observed against 27 non-*C. tropicalis* strains. In clinical validation using 128 specimens, the assay showed 100.0% sensitivity and 97.3% specificity compared with a composite reference standard, with near-perfect agreement (κ = 0.968). The entire process can be completed within approximately 53 min, and results can be visualized under blue light without sophisticated instrumentation. These findings indicate that the MCDA-CRISPR/Cas12a assay is a rapid, sensitive, and specific diagnostic tool for *C. tropicalis* detection, particularly suitable for resource-limited settings.

## Data Availability

The original contributions presented in the study are included in the article/**Supplementary Material**. Further inquiries can be directed to the corresponding authors.
