# Supplementary material for: Rapid and ultrasensitive detection of Candida tropicalis using multiple cross-displacement amplification combined with CRISPR/Cas12a
Source: Front Cell Infect Microbiol. 2026 Jul 30;16:1796886. doi: 10.3389/fcimb.2026.1796886 (PMC13467988; doi:10.3389/fcimb.2026.1796886)
Supplement: Supplementary file 1 [file DataSheet1.docx]

Supplementary Material

Rapid and ultrasensitive detection of *Candida tropicalis* using multiple cross-displacement amplification combined with CRISPR/Cas12a

**Rui Ye ^1，2，3^*^†^*, Mao Liu ^1，2，3^*^†^*, Xujian Zhang ^2^, Qi Liang ^1，2，3^, Yumei Cao ^2^, Chuiyu Zhu ^2^, Honglan Yu^2*^, Shu Zhang ^1，3*^, Yu Wang^1，2*^**

*^1^ Department of Basic Clinical Laboratory Medicine, School of Clinical Laboratory Science, Guizhou Medical University, Guiyang, 550001, Guizhou, P. R. China.*

*^2^ Department of Clinical laboratory, The First People’s Hospital of Guiyang, Guiyang, 550004, Guizhou, P. R. China.*

*^3^ Center for Clinical Laboratories, The Affiliated Hospital of Guizhou Medical University, Guiyang, 550001, Guizhou, P. R. China.*

***^†^These authors have contributed equally to this work.***

***Correspondence:** *Yu Wang: wangzhongyuwy@163.com;*

*Shu Zhang: zhangshu@gmc.edu.cn;*

*Honglan Yu:1049251463@qq.com*

**Inventory of Supplementary Information:**

1. **Supplementary tables**

Table S1. The primers, probe and crRNA used in the current study

Table S2. Strains used for specificity evaluation in this study

Table S3. Summary table of repetitive validation results

Table S4. Raw results of the three detection methods

Table S5. Complete cross-tabulation of all three methods

Table S6. Comparison of key characteristics of MCDA, LAMP, and RPA isothermal amplification techniques.

2. **Supplementary Figures**

Figure S1. Chematic diagram of the primer sequences for the MCDA reaction of *Candida tropicalis*

Figure S2. Screening and confirmation of *Candida tropicalis* standard strain

**1 Supplementary tables**

**Table S1. The primers, probe and crRNA used in the current study**

| **MCDA Primers / Probe / crRNA** | **Sequence and modification (5′-3′) ^b^** | **Length^c^** |
| --- | --- | --- |
| F1 | CGATACGTAATATGAATTGCAGAT | 24 nt |
| F2 | CCTACCTGATTTGAGGTCAA | 20 nt |
| CP1 ^a^ | CAGGCATGCCCTTTGGAATA-**TTTC**-GTGAATCATCGAATTTTTGAACG | 47 nt |
| CP2 | AACGTGGAAACTTATTTTAAGCGACATTGTGGTGGCCACTAGC | 44 nt |
| C1 | CAGGCATGCCCTTTGGAATA | 20 nt |
| C2 | AACGTGGAAACTTATTTTAAGCGAC | 25 nt |
| D1 | CCAAAGGGCGCAATGTG | 17 nt |
| D2 | AGGTTTATCCAAAAACGCTT | 20 nt |
| R1 | AGGGAGAAATGACGCTCAA | 19 nt |
| R2 | TGAGCAATACGCTAGGTT | 18 nt |
| crRNA | UAAUUUCUACUAAGUGUAGAU--GUGAAUCAUCGAAUUUUUGAACG | 44 mer |
| Probe | FAM-TATTATTATTATTATTT-BHQ1 | 17 mer |

^a^ CP1, The substitution primer modified by "TTTC" in the *C. tropicalis*-MCDA-CRISPR/Cas12a assay.

^b^ nt, nucleitide.

^c^ mer, monomeric unit.

**Table S2. The strains used for specific screeningin this study**

| **Strains** | **Source of strains** | **No. of strains** | ***C. tropicalis*-MCDA-CRISPR/Cas12a** |
| --- | --- | --- | --- |
| *Candida tropicalis* | NCCL^a^ | 2 | P |
| *Candida tropicalis* | GZCCL^b^ | 2 | P |
| *Candida tropicalis* | Isolated strains (GFPH)^c^ | 8 | P |
| *Candida auris* | NCCL | 1 | N |
| *Candida krusei* | GZCCL | 1 | N |
| *Candida glabrata* | NCCL | 1 | N |
| *Candida parapsilosis* | NCCL | 1 | N |
| *Candida albicans* | ATCC 10231 | 1 | N |
| *Candida lusitaniae* | NCCL | 1 | N |
| *Corynebacterium gladioliforme* | GZCCL | 1 | N |
| *Lichtheimia ramosa* | Isolated strains (GFPH) | 1 | N |
| *Talaromyces marneffei* | Isolated strains (GFPH) | 1 | N |
| *Aspergillus flavus* | Isolated strains (GFPH) | 1 | N |
| *Aspergillus fumigatus* | Isolated strains (GFPH) | 1 | N |
| *Cryptococcus neoformans* | NCCL | 1 | N |
| *Saccharomyces cerevisiae* | NCCL | 1 | N |
| *Klebsiella pneumoniae* | Isolated strains (GFPH) | 1 | N |
| *Pseudomonas aeruginosa* | ATCC 27853 | 1 | N |
| *Acinetobacter baumannii* | Isolated strains (GFPH) | 1 | N |
| *Staphylococcus aureus* | ATCC 29213 | 1 | N |
| *Escherichia coli* | ATCC 25922 | 1 | N |
| *Streptococcus pneumoniae* | ATCC 49619 | 1 | N |
| *Haemophilus influenzae* | ATCC 10211 | 1 | N |
| *Salmonella typhimurium* | ATCC 12022 | 1 | N |
| *Enterococcus faecium* | Isolated strains (GFPH) | 1 | N |
| *Mycoplasma pneumoniae* | Isolated strains (GFPH) | 1 | N |
| *Adenovirus* | Isolated strains (GFPH) | 1 | N |
| *Respiratory syncytial virus* | Isolated strains (GFPH) | 1 | N |
| *Rhinovirus* | Isolated strains (GFPH) | 1 | N |
| *SARS-CoV-2* | Isolated strains (GFPH) | 1 | N |

^a^NCCL: National Center for Clinical Laboratories

^b^GZCCL: Guizhou Center for Clinical Laboratories

^c^GFPH: *The First People’s Hospital of Guiyang*

**Table S3. Summary table of repetitive validation results**

| **Sample number** | **Testing type** | **Repeat 1** | **Repeat 2** | **Repeat 3** | **Consistency judgment**^a^ |
| --- | --- | --- | --- | --- | --- |
| Intra 1 | Intra-assay analysis | Pos | Pos | Pos | **3/3（100%）** |
| Intra 2 | Intra-assay analysis | Pos | Pos | Pos | **3/3（100%）** |
| Intra 3 | Intra-assay analysis | Pos | Pos | Pos | **3/3（100%）** |
| Intra 4 | Intra-assay analysis | Pos | Pos | Pos | **3/3（100%）** |
| Intra 5 | Intra-assay analysis | Pos | Pos | Pos | **3/3（100%）** |
| Intra 6 | Intra-assay analysis | Neg | Neg | Neg | **3/3（100%）** |
| Inter 1 | Inter-assay analysis | Pos | Pos | Pos | **3/3（100%）** |
| Inter 2 | Inter-assay analysis | Pos | Pos | Pos | **3/3（100%）** |
| Inter 3 | Inter-assay analysis | Neg | Neg | Neg | **3/3（100%）** |
| Inter 4 | Inter-assay analysis | Neg | Neg | Neg | **3/3（100%）** |
| Inter 5 | Inter-assay analysis | Pos | Pos | Pos | **3/3（100%）** |
| Inter 6 | Inter-assay analysis | Pos | Pos | Pos | **3/3（100%）** |

^a^Consistency judgment = (Total number of consistent results / Total number of tests) × 100% .

**Table S4. Raw results of the three detection methods**

| **Method** | **Positive** | **Negative** | **Total** |
| --- | --- | --- | --- |
| *C. tropicalis*-MCDA-CRISPR/Cas12a assay | 56 | 72 | 128 |
| Fungal culture | 51 | 77 | 128 |
| Multiplex PCR with capillary electrophoresis | 54 | 74 | 128 |

**Table S4.** Raw results of the three detection methods for 128 clinical specimens. Positivity was defined based on each method's own criteria: visible growth on culture plates for fungal culture, amplification peaks in capillary electrophoresis for multiplex PCR, and fluorescent signal or blue-light visual readout for the MCDA-CRISPR/Cas12a assay. CRS, composite reference standard.

**Table S5. Complete cross-tabulation of all three methods**

| **Group** | **Culture** | **PCR** | **MCDA** | **Number of Cases** |
| --- | --- | --- | --- | --- |
| A | **+** | **+** | **+** | 51 |
| B | **+** | **+** | **−** | 0 |
| C | **+** | **−** | **+** | 0 |
| D | **+** | **−** | **−** | 0 |
| E | **−** | **+** | **+** | 3 |
| F | **−** | **+** | **−** | 0 |
| G | **−** | **−** | **+** | 2 |
| H | **−** | **−** | **−** | 72 |
| **Total** |  |  |  | **128** |

**Table S5.** Complete cross‑tabulation of the three methods for 128 clinical specimens. Groups A–H represent all possible combinations of positive (+) and negative (−) results among fungal culture, multiplex PCR, and the C. tropicalis-MCDA-CRISPR/Cas12a assay. Group A: all three methods positive; Group E: positive by PCR and MCDA only; Group G: positive by MCDA only; Group H: all three methods negative. The composite reference standard (CRS) was defined as positive if either culture or PCR was positive, and negative only when both were negative. Detailed raw results are provided in Table S4.

**Table S6. Comparison of key characteristics of MCDA, LAMP, and RPA isothermal amplification techniques**

| **Parameter** | **MCDA** | **RPA** | **LAMP** |
| --- | --- | --- | --- |
| Year introduced | 2015 | 2006 | 2000 |
| Template | DNA and RNA | DNA and RNA | DNA and RNA |
| Detection time | ~40 min | 25–40 min | ~60 min |
| Number of primers | 10 | 2 | 4–6 |
| Reaction temperature | 60–65°C | 37–42°C | 60–65°C |
| Enzymes for amplification | *Bst* DNA polymerase | Recombinase, *Bst* DNA polymerase, and single-stranded DNA-binding protein | *Bst* DNA polymerase |
| Cost | Low (single-enzyme system) | High (multi-enzyme complex) | Low (single-enzyme system) |
| Advantages | High specificity, low contamination risk, CRISPR-compatible | Mild temperature, no heating, fast | Rapid, low-cost, well-established |
| Limitations | Primer design complexity; requires 65°C heater | High reagent cost, environmentally sensitive | Non-specific amplification, false-positive risk |

Abbreviations: MCDA, multiple cross displacement amplification; LAMP, loop-mediated isothermal amplification; RPA, recombinase polymerase amplification.

**2 Supplementary Figures**

**Figure S1.Chematic diagram of the primer sequences for the MCDA reaction of *Candida tropicalis***

Based on the ITS II gene sequence of *C. tropicalis*, specific primers and gRNA for the *C. tropicalis.* The positions of the MCDA primers are marked with underlines. The right and left arrows represent the forward and reverse strand sequences used in this study.

M 1 2 3 4 5

1,5

2

3

4

**Figure S2. Screening and confirmation of *Candida tropicalis* standard strains**

M, Marker; Line 1, *Candida albicans*; Line 2: The second inter-laboratory quality assessment of the microbial group conducted by National Quality Control Center in 2022. Number 202211, *Candida tropicalis*; Line 3: The first inter-laboratory quality assessment of the microbial group conducted by Guizhou Provincial Quality Control Center in 2023. Number 20233002, *Candida tropicalis*; Line 4: The second inter-laboratory quality assessment of the microbial group conducted by National Quality Control Center in 2024. Number 202408, *Candida tropicalis*; Line 5, Negative control. The standard strain used in the optimization of this research system is 202408.
